# Ambulatory Blood Pressure Patterns and Left Ventricular Mass Index in Tanzanian Adults Living with and without HIV

**DOI:** 10.5334/gh.1542

**Published:** 2026-03-24

**Authors:** Megan Willkens, Benson Issarow, Anthony O. Etyang, Salama Fadhil, Cody Cichowitz, Philip Ayieko, Godfrey Kisigo, Sara Venkatraman, Ana C. Krieger, Richard Devereux, Paul Muntner, Myung Hee Lee, Saidi Kapiga, Robert Peck

**Affiliations:** 1 Center for Global Health, Department of Medicine, Weill Cornell Medicine, New York, New York, USA; 2 Mwanza Intervention Trials Unit, National Institute of Medical Research, Mwanza, Tanzania; 3 Department of Epidemiology and Demography, KEMRI-Wellcome Trust Research Programme, Kilifi, Kenya; 4 Division of Cardiology, Department of Medicine, University of California San Francisco, San Francisco, California, USA; 5 Department of Infectious Disease Epidemiology, London School of Hygiene and Tropical Medicine, London, United Kingdom; 6 Weill Cornell Center for Sleep Medicine, Department of Medicine, Weill Cornell Medical College, New York, New York, USA; 7 Division of Cardiology, Department of Medicine, Weill Cornell Medicine, New York, New York, USA; 8 Department of Epidemiology, School of Public Health, University of Alabama at Birmingham, Birmingham, Alabama, USA; 9 Perisphere Real World Evidence, Austin, Texas, USA

**Keywords:** people with HIV, ambulatory blood pressure monitoring, Northwest Tanzania, hypertension, left ventricular mass index, HIV clinic

## Abstract

**Background:**

People with HIV (PWH) experience an increased risk of cardiovascular disease (CVD). Small studies suggest that PWH have less nocturnal dipping, but the clinical importance of this difference is not known.

**Objectives:**

The aim of this study was to compare ambulatory blood pressure monitoring (ABPM) parameters by HIV status and quantify the effect of ABPM parameters on left ventricular mass index (LVMI) in PWH and people without HIV (PWoH).

**Methods:**

We conducted a cross-sectional analysis of enrollment and one-month data from PWH and PWoH from the Mwanza HIV&CVD cohort study. Unattended, automated office BP and ABPM measurements were conducted at two time points and averaged. Regression models, adjusted for CVD risk factors, were used to compare ABPM parameters by HIV status. Twenty-four-hour ABPM patterns were displayed using time series analysis. The association between ABPM parameters and LVMI was tested, controlling for office BP. Analyses were stratified by hypertension status.

**Results and conclusions:**

Of the 999 participants enrolled, 959 (96.0%) had at least one complete ABPM measurement (483 PWH/476 PWoH). Office BP was higher in PWoH compared to PWH but nocturnal BPs were similar. Nocturnal dipping percentages were lower in PWH. The prevalence of diastolic non-dipping was 41% in PWH compared to 28% in PWoH (incidence rate ratio 1.42 [1.20,1.69]). Differences in dipping patterns and discrepancies between office and ABPM were apparent in time series analysis. Asleep systolic BP was independently associated with LVMI even after adjusting for office systolic BP in PWH, but not in PWoH. PWH had less nocturnal dipping than PWoH and asleep BP was independently associated with higher LVMI in PWH only. These findings suggest that office BP might underestimate the true BP-related CVD risk in PWH.

## Introduction

People with HIV (PWH) have twice as high a risk of cardiovascular disease (CVD) and CVD-related mortality as people without HIV (PWoH) of similar age (1,2). This increased risk of CVD in PWH is present even after adjusting for traditional risk factors (3,4). Some evidence suggests that elevated blood pressure (BP) and hypertension, based on BP measured in an office setting, are more strongly associated with CVD risk in PWH than in PWoH (5,6).

Ambulatory blood pressure monitoring (ABPM) has a stronger association with risk of CVD events compared to BP measured in an office setting (7). In the general population, higher nighttime systolic BP estimated by ABPM is a strong CVD and mortality risk factor, even after adjusting for office systolic BP (8,9,10). Non-dipping BP, defined as a <10% decrease in BP from awake to asleep, may also be a CVD risk factor (9,12,13).

Small studies of ABPM in PWH suggest that PWH experience less nocturnal dipping of BP than PWoH (14,15). Although the value of using ABPM to guide antihypertensive treatment in the general population is becoming more widely recognized, few data are available to guide its use in PWH (14,16). Therefore, we are studying the relationship between ABPM and pre-clinical CVD in a study of 499 PWH and 500 PWoH in Tanzania. From this study, we recently reported that office BP was lower in PWH but the prevalence of sustained hypertension and masked hypertension were similar, with a prevalence of ~25% in both PWH and PWoH, which may be explained by relatively higher asleep BP in PWH (11).

The goal of the current study was to determine (1) the association between HIV status and average office, awake, and asleep BP and non-dipping BP; (2) 24-hour BP trajectories in PWH and PWoH; and (3) the associations of average awake and asleep BP with left ventricular mass index (LVMI) in the two groups. We hypothesized that office, awake, and asleep BP would be higher and non-dipping BP would be more common in people with versus without HIV. In addition, higher nighttime BP, relative to office BP, may partially explain the higher-than-expected left ventricular mass in PWH.

## Methods

### Study design and location

We conducted a cross-sectional analysis of enrollment and one-month data from PWH and PWoH from the Mwanza HIV&CVD cohort study. PWH and PWoH were enrolled from three publicly funded HIV clinics in Mwanza, Tanzania, a city on the Northern shore of Lake Victoria.

### Study population

We enrolled PWH and treatment supporters who were PWoH. According to Tanzanian national HIV guidelines, every PWH must have a ‘treatment supporter’ whose role is to promote treatment adherence (17). We have previously shown that PWH and their treatment supporters have similar sociodemographic characteristics since they are drawn from the same source population (18,19,20). We selected control participants from among all treatment supporters attending the HIV clinic during the study period without individual matching. We selected the control group of PWoH to closely resemble the group of PWH according to age and sex, using directed enrollment of treatment supporters to balance the two groups. PWH and PWoH were eligible if they were 30 years of age or older. To be eligible for the study, PWH had to have been taking antiretroviral therapy (ART) for at least 180 days prior to enrollment. Exclusion criteria for both groups were serious illness or trauma requiring systemic treatment or hospitalization in the past 30 days, current pregnancy, any psychiatric or psychological conditions, and nocturnal work that would interfere with the study procedures.

### General study procedures

At enrollment, participants were administered study questionnaires and underwent physical examination; BP measurements were taken in an office setting and ABPM was conducted. At one month, office BP measurements and ABPM were repeated. Participants were started on antihypertensive medication after the one-month visit if they were confirmed to have hypertension (21).

Information on CVD risk factors and demographic and socioeconomic data were collected using a questionnaire that had been translated into Kiswahili, validated in East Africa, and used by our research team in Tanzania for the past decade. This questionnaire is part of the World Health Organization’s STEPwise Surveillance (STEPS) procedures and includes questions about use of antihypertensive medication within the last week. Height, weight, and waist circumference were measured, and a blood sample was collected during each study visit. Body mass index (BMI) was calculated, and fasting blood glucose level was measured. Diabetes was defined as fasting blood glucose ≥7 mmol/L or currently taking medication to treat diabetes.

### Cardiovascular and sleep measurements

Unattended, automated office BP was measured at enrollment and one-month timepoints using an OMRON HBP-1300 (OMROM Healthcare, Kyoto, Japan) BP monitor according to recommendations for clinical research (22). Participants rested for five minutes, sitting with their back supported, legs uncrossed, and feet flat on the floor. A research assistant informed each participant of the procedure for BP measurement, measured the participant’s arm circumference and attached the appropriately-sized BP cuff, initiated the machine measurement, and then left the participant alone in the room. Three successive, unattended, automated BP measurements were taken on the non-dominant arm with a 30-second interval between measurements. Average BP was calculated from the second and third measurements.

ABPM was performed for 24 hours using the Mobil-O-Graph device (IEM GmbH, Germany) at enrollment and one month later. A BP cuff was placed on the participant’s non-dominant arm and the recorder was secured around the waist. The ABPM was programmed to measure BP every 30 minutes while awake and 60 minutes during sleep. Sleep times to inform awake and sleep periods during ABPM measurement were quantified using 24-hour actigraphy with the Micro Motionlogger® Watch (Ambulatory Monitoring Inc, Ardsley, NY), and complemented by self-report.

An ABPM recording was deemed complete if >70% of all scheduled systolic and diastolic blood pressure measurements were successful, with at least three valid recordings at night. Participants with at least one complete ABPM recording, at baseline or one month later, were included in the analysis. For those with complete ABPM measurements at both time points, the average of the two measurements was used. If two ABPM measurements were used, their respective office BP measurements were averaged. For those with only one complete ABPM recording, only the office BP measurement taken at the same time point was used. Mean awake and asleep BPs were calculated as the average of measurements taken while the participant was awake and asleep, respectively. Mean 24-hour BP was calculated using mean awake and asleep BP weighted to the amount of time the participant was awake and asleep. Hypertension was defined according to the ABPM results as 24-hour average systolic/diastolic BP ≥130/80 mm Hg, and/or awake average systolic/diastolic BP ≥135/85 mmHg, and/or asleep average systolic/diastolic BP ≥120/70 mmHg (23,24). Nocturnal dipping percentage, percent change in BP from awake to asleep, was calculated as the average awake minus the average asleep BP divided by the average awake BP. Non-dipping was defined as a <10% dipping percentage (19).

Echocardiography was performed by two technologists credentialed in echocardiography following the American Society of Echocardiography guidelines at the enrollment visit (25). The methods used for echocardiography have previously been reported (20). Left ventricular mass was calculated using the Devereux formula and was indexed for body surface area (LVMI) for analysis as a continuous variable (26).

### Analysis

Medians and proportions were used to summarize baseline characteristics, and means and standard deviations were used to summarize BP levels by HIV status. ABPM outcomes of interest included systolic and diastolic mean asleep BP, mean awake BP, and nocturnal dipping. To quantify the differences between office and ambulatory BP, we subtracted office BP from awake and sleep ABPM measurements. To compare the rate of non-dipping by HIV status, we used Poisson regression. For comparing the prevalence of non-dipping between PWH and PWoH groups by hypertension status, we used two-sample proportions tests. To confirm the rate of non-dipping, we examined persistent non-dipping on both measurements for participants with ABPM at both baseline and one month. We used linear regression models to estimate the difference in BP levels by HIV status. We performed linear regression to test the association between BP parameters and LVMI. All regression models were adjusted for age, sex, BMI, educational attainment, smoking, alcohol use, taking antihypertensive medication, and hemoglobin. In the final model for mean awake/asleep BP and LVMI, we adjusted for office systolic BP and stratified by HIV and hypertension status to determine whether the ABPM parameters added incremental information beyond what was provided by office BP.

We also conducted a time series analysis of the ABPM data to visualize BP trajectories over 24 hours in PWH compared to PWoH. Time series analyses were stratified by hypertension status since BP trajectories differed substantially between those with and without hypertension. For the time series analysis, individual trajectories were smoothed using a centered three-point moving average. Each individual trajectory was displayed as a string plot with a solid line overlaid to represent the average time series across participants. This average time series was calculated by taking the average ABPM at each time point between 10 AM on the first day of data collection and 8 AM the following day. We fit a linear mixed-effects model of smoothed diastolic BP as a function of time, HIV status, and hypertension status as fixed effects and a participant-specific random effect. Time was treated as a factor variable and HIV and hypertension statuses were binary indicators. The coefficient on HIV status represents the average difference in diastolic BP between PWH and PWoH across all time points, holding hypertension status fixed.

We conducted two sensitivity analyses. First, we repeated the above analyses restricted to participants with >70% valid ABPM measurements and at least 20 awake and 7 asleep measurements (N = 812). Second, we repeated the above analyses restricted to participants who were not taking antihypertensives (N = 936).

### Ethical approval

This study was approved by the institutional review boards at Weill Cornell Medicine and the Tanzanian National Institute of Medical Research. All participants provided written informed consent in Kiswahili.

## Results

### Study population and baseline characteristics

Of the 999 participants enrolled between March 2022 and May 2023, 959 (96.0%) had at least one complete ABPM recording, including 483 of 499 (96.6%) PWH and 476 of 500 (95.4%) PWoH. The median age was higher among PWH compared to PWoH (46 [25–75 percentiles 39–50] versus 43 (36–50) years) and 69.1% and 70.4% of PWH and PWoH were female, respectively (Table 1). The prevalence of hypertension was 51.9%. The majority of participants had seven years or fewer of education (80.7%). Only 2.3% were currently taking antihypertensive medication and all of these were on single-drug therapy with a calcium channel blocker or thiazide diuretic. All participants with PWH were on the standard, first-line antiretroviral regimen for Tanzania, including a combination of two nucleoside reverse transcriptase inhibitors (tenofivir and lamivudine) and one integrase inhibitor (dolutegravir) combined into a single pill. At the time of enrollment, participants had been on ART for an average of 7.8 (SD 4.3) years. All PWH had suppressed viral loads.

**Table 1:** Characteristics of 959 participants with and without HIV.


	PWH (N = 483) MEDIAN [25–75 PERCENTILES]/N (%)	PWOH (N = 476) MEDIAN [25–75 PERCENTILES]/N (%)

**Age** in years	46 [39–50]	43 [36–50]

**Female sex**	340 (70.4)	329 (69.1)

**Education level**		

Primary school or less	411 (85.1)	363 (76.3)

Complete secondary school	59 (12.2)	90 (18.9)

University/college	13 (2.7)	23 (4.8)

**Low income** (<1.90 USD/day)	355 (73.5)	344 (72.3)

**Mode of transport**		

Private vehicle	70 (14.5)	57 (12.0)

Public transport	251 (52.0)	241 (50.6)

Walking/cycling	162 (33.5)	178 (37.4)

**Manual labor**	156 (32.3)	154 (32.4)

**Current tobacco use**	20 (4.1)	35 (7.4)

**Current alcohol use**	149 (30.8)	138 (29.0)

**Hypertension**	248 (51.3)	250 (52.5)

On hypertension medication*	9 (1.9)	14 (2.9)

**LVMI** (g/m^2^) (mean (SD))	65.9 [55.0–79.6]	65.4 [54.8–78.4]

**BMI**		

Underweight (<18.5 kg/m^2^)	57 (11.8)	65 (13.7)

Normal (18.5–24.9 kg/m^2^)	273 (56.5)	245 (51.5)

Overweight/Obese (≥25 kg/m^2^)	153 (31.7)	166 (34.9)

**Diabetes**	9 (1.8)	4 (0.8)

**Waist circumference** (cm)	83.4 [75.6–93.6]	83.8 [76.5–94.1]

**Hemoglobin** (g/dl)	13.3 [11.6–14.8]	13.7 [12.5–15.0]

**Chronic kidney disease**	31 (6.4)	5 (1.1)

**CD4+ T-cell count** (cells/mm^3^)***	717 [539–953.5]	N/A


*All participants on hypertension medication were on hydrochlorothiazide monotherapy. **Defined according to eGFR <60 mL/min/1.73 m^2^. ***N/A = not applicable.

### Blood pressure parameters and HIV

After multivariable adjustment, office systolic and diastolic BP was lower in PWH compared to PWoH controls (–2.8 [95% CI –4.8, –0.9] mmHg, and –1.7 [–3.1, –0.2] mmHg, respectively) (Table 2). Systolic and diastolic awake mean BP was lower in PWH compared to PWoH (–1.6 [–3.1, –0.1] mmHg and –0.9 [–2.1, 0.2] mmHg, respectively). There was no evidence of a difference in mean sleep BP between the two groups. For nocturnal dipping percentage, both systolic and diastolic dipping percentages were lower in PWH (1.0 [0.3, 1.7] and 1.3 [0.4, 2.2], respectively). The differences between awake mean systolic and diastolic BP as compared to office BP in PWH versus PWoH were 0.9 [–0.4, 2.1] mmHg, p = 0.167 and 0.9 [0.1, 1.8] mmHg, p = 0.04, respectively.

**Table 2:** Office, awake, and asleep blood pressure and blood pressure dipping for participants with and without HIV.


	PWH (N = 483) MEAN (SD)	PWOH (N = 476) MEAN (SD)	ADJUSTED DIFFERENCE [95% CI]	p-VALUE

*BP parameters from office and ambulatory BP measurements*				

**Office systolic BP**, mmHg	113 (16.2)	116 (16.2)	–2.8 [–4.8, –0.9]	0.005

**Office diastolic BP**, mmHg	69 (12.1)	71 (11.2)	–1.7 [–3.1, –0.2]	0.022

**Awake mean systolic BP**, mmHg	122 (12.1)	124 (12.4)	–1.6 [–3.1, –0.1]	0.039

**Awake mean diastolic BP**, mmHg	80 (10.0)	82 (9.4)	–0.9 [–2.1, 0.2]	0.118

**Asleep mean systolic BP**, mmHg	114 (12.6)	114 (12.4)	–0.2 [–1.8, 1.4]	0.805

**Asleep mean diastolic BP**, mmHg	70.7 (9.6)	70.5 (9.5)	0.2 [–1.0, 1.4]	0.781

**24-h mean systolic BP**, mmHg	119 (11.9)	120 (11.9)	–0.6 [1.8, 0.5]	0.270

**24-h mean diastolic BP**, mmHg	77 (9.5)	78 (9.0)	0.2 [–1.0, 1.4]	0.781

**Nocturnal dipping: systolic BP** (%)	–6.4 (5.6)	–7.5 (5.6)	1.0 [0.3, 1.7]	0.005

**Nocturnal dipping: diastolic BP** (%)	–11.7 (6.9)	–13.5 (7.1)	1.3 [0.4, 2.2]	0.005

	**PWH (N = 483) MEAN (SD)**	**PWOH (N = 476) MEAN (SD)**	**UNADJUSTED DIFFERENCE [95% CI]**	**p-VALUE**

*Within-participant differences between ambulatory and office BP measurements*				

**Awake mean systolic BP minus office BP difference**, mmHg	8.3 (9.5)	7.4 (10.1)	0.9 [–0.4, 2.1]	0.167

**Awake mean diastolic BP minus office BP difference**, mmHg	11.2 (7.3)	10.2 (6.6)	0.9 [0.1, 1.8]	0.04

**Asleep mean systolic BP vs. office BP difference**, mmHg	0.4 (11.1)	–2.0 (11.7)	2.4 [1.0, 3.9]	0.001

**Asleep mean diastolic BP vs. office BP difference**, mmHg	1.7 (8.3)	–0.9 (7.9)	2.6 [1.5, 3.6]	<0.001


This table displays the relationship between BP parameters and HIV status both with raw averages and differences for BP parameters. The differences in the upper portion of the table are adjusted for age, sex, BMI, tobacco use, alcohol use, taking antihypertensives, and hemoglobin. We report unadjusted results for within-participant differences (bottom portion) because we do not expect any bias due to covariates. *SD = Standard deviation.

### Time series analysis of ABPM results

**Figure 1:**
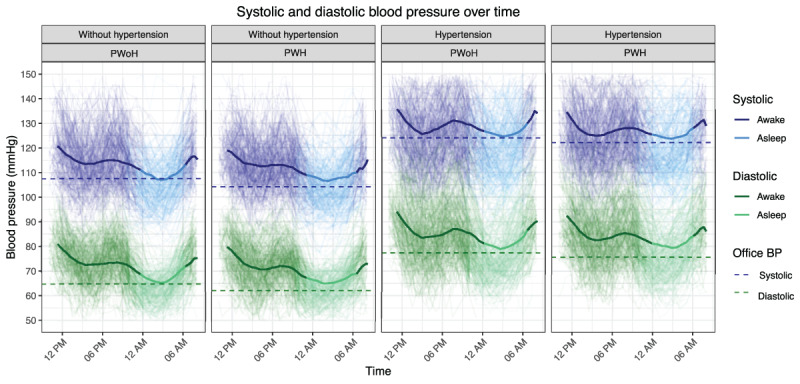
Average systolic (top) and diastolic (bottom) ambulatory blood pressure monitoring in PWoH (left) and PWH (right). Background lines display each participant’s ABPM trajectory, with the darker shade denoting their measurements while awake and the lighter shade denoting measurements while asleep. Individual trajectories are smoothed using a centered three-point moving average. The solid line overlaid represents the average time series across participants (calculated by taking the average ABPM at each time point between approximately 10 AM on the first day of data collection and 8 AM on the following day), and the horizontal dotted line represents the average office systolic and diastolic blood pressure measurements.

Time series analysis of systolic and diastolic BP trajectories over 24 hours in PWH and PWoH visualized differences in non-dipping patterns between PWH and PWoH (Figure 1). The average time series trajectories also displayed differences between groups in ambulatory relative to office BPs. These differences were seen both in participants with and without hypertension. In the linear mixed-effects model, the average diffierence in diastolic BP by HIV status was –1.11 ([95% CI –2.09,–0.13], p-value = 0.027) and statistically significant at a 0.05 level, indicating that PWH have diastolic BP that is on average 1.11 mmHg lower than that of their PWoH counterparts.

### Non-dipping and HIV

**Figure 2:**
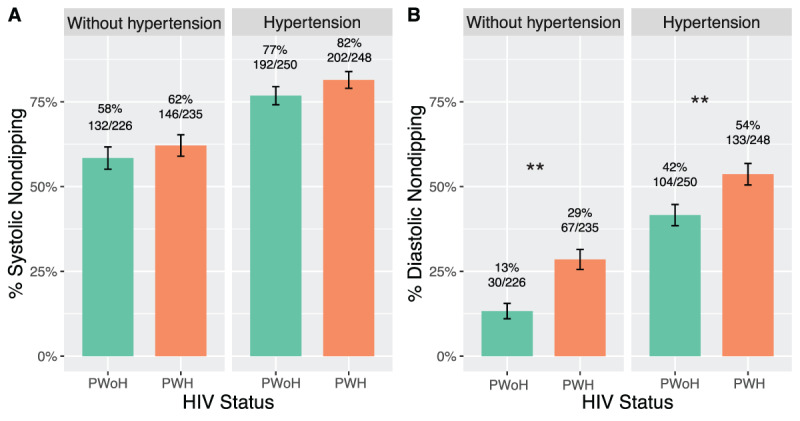
Systolic and diastolic non-dipping for PWH and PWoH, stratified by hypertension status. ** Indicates p-values less than 0.01.

The prevalence of systolic non-dipping was 72% in PWH and 68.3% in PWoH (incidence rate ratio (IRR) 1.04 [95% CI 0.96,1.13]). However, the rate of diastolic non-dipping was 1.42 times higher in PWH (41%) compared to PWoH (28%) with adjustment for traditional CVD risk factors (age, sex, BMI, current smoking, alcohol use, taking antihypertensive medication, and hemoglobin) (IRR 1.42 [1.20,1.69]). By hypertension status, the prevalence of systolic non-dipping was 60% in participants without hypertension and 80% in participants with hypertension (Figure 2). Among PWH versus PWoH, the prevalence of systolic non-dipping was 82% vs. 77% in those with hypertension and 62% vs. 58% in those without hypertension.The prevalence of diastolic non-dipping was 54% vs. 42% in PWH with hypertension and 29% vs. 13% in PWH without hypertension (Figure 2; **Table S1**). Of the 814 participants with complete ABPM measurements at both time points, 56% of PWH had persistent systolic non-dipping compared to 48% of PWoH and 24% of PWH had persistent diastolic non-dipping compared to 13% of PWoH.

### Blood pressure parameters and left ventricular mass index

Median LVMI was 65.9 [25–75 percentiles 55.0–79.6] g/m^2^ in PWH versus 65.4 [54.8–78.4] g/m^2^ in PWoH. Office, awake, and asleep systolic and diastolic BP were associated with increased LVMI for both PWH and PWoH (**Figure S1**), after adjusting for covariates.

**Figure 3:**
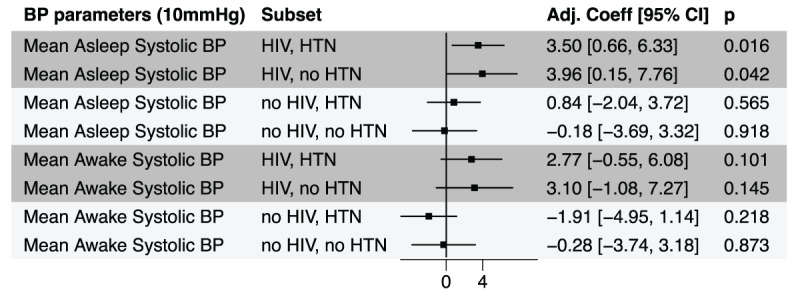
Adjusted difference in LVMI in g/m2 for higher awake and asleep blood pressure for people with and without HIV and with and without hypertension. Adj Coeff = regression coefficient for each ABPM parameter in models adjusted for office systolic blood pressure and traditional CVD risk factors including age, sex, BMI, education, tobacco use, alcohol use, and antihypertensive medication use. Among people with HIV and hypertension, a 10 mmHg increase in mean asleep systolic blood pressure was associated with a 3.5 g/m^2^ increase in LVMI.

For PWoH with and without hypertension, mean asleep and awake systolic BP was not associated with LVMI after accounting for office systolic BP (Figure 3). For PWH, after adjustment for office BP, asleep systolic BP was associated with LVMI in participants both with and without hypertension. For PWH, higher awake systolic BP was not associated with LVMI among those with and without hypertension.

### Sensitivity analyses

For the first sensitivity analysis, 812 participants (404 PWH and 408 PWoH) had at least one ABPM recording with 20 awake measurements and 7 asleep measurements (**Supplemental Material**). For PWH, asleep systolic BP was associated with LVMI in participants both with and without hypertension; each 10 mmHg increase in asleep systolic BP was associated with a 4.8 [2.0,7.6] g/m^2^ and 3.6 [–0.8,8.2] g/m^2^ higher LVMI in PWH with and without hypertension, respectively.

For the second sensitivity analysis, 936 participants (474 PWH and 462 PWoH) had at least one recording meeting the standard quality definition for ABPM (>70% valid and three asleep measurements) and were not currently taking antihypertensives. Asleep systolic BP was again associated with LVMI among PWH only, where each 10 mmHg increase in asleep systolic BP was associated with a 3.4 [0.5,6.3] g/m^2^ and 3.9 [0.1,7.7] g/m^2^ higher LVMI in PWH with and without hypertension, respectively.

## Discussion

There are three main findings from this large comparative cohort of PWH and PWoH with two ABPM recordings and rigorous measurement of office BP. First, PWH demonstrate less nocturnal dipping than PWoH. Second, office BP appears to underestimate asleep BP in PWH. Third, asleep BP measured by ABPM was associated with higher left ventricular mass even after adjustment for office BP.

PWH had less nocturnal dipping than PWoH in the current study. The lower nocturnal dipping was demonstrated by a lower systolic and diastolic dipping percentage, the visualization of less nocturnal dipping in our time-series analysis, and the higher prevalence of diastolic nocturnal non-dipping as a binary variable. The prevalence of systolic nocturnal non-dipping was high in all study groups, which is consistent with other studies in Africa (27). These findings confirm and extend the findings of multiple smaller cross-sectional studies from PWH in both high and low-income countries (14,15). Nocturnal non-dipping of BP may itself be an independent risk factor for CVD events (28). Although this point is widely debated (29), nocturnal non-dipping remains important for its relative effect on nocturnal BP.

Prior studies have shown that elevated BP and hypertension are associated with an even greater risk of CVD in PWH as compared to PWoH. Our findings may help to explain the results of these prior studies. For example, in a longitudinal analysis of 4027 CVD events in the MarketScan database, the hazard ratio for acute myocardial infarction associated with hypertension was 1.70 [1.44, 2.01] among PWH versus 1.35 [1.22,1.51] among PWoH (p-value for interaction = 0.017) (5). A study in the Veterans Aging Cohort Study Virtual Cohort revealed similar findings (6). In addition, we have previously demonstrated that HIV interacts with pre-hypertension to increase the risk of microvascular disease (30). All these findings could be explained by our current observation that elevated office BP in PWH is associated with an even higher asleep BP than would be expected in PWoH. In the context of prior studies, our observation suggests PWH might require BP treatment to a lower office BP target, as would be offered to people at an elevated risk of future cardiovascular events. Such a strategy would be consistent with what is already recommended for other CVD risk-enhancing conditions such as diabetes and kidney disease (31).

The lesser nocturnal dipping in PWH might explain our first finding, the relatively higher asleep BP compared to office blood pressure in PWH. Examining our time series analysis, it appears that the lesser nocturnal dip in PWH explains part of the observation that asleep blood pressure remains higher than office BP in PWH. Alternative explanations might include (1) a greater familiarity with the office setting in PWH resulting in a lower office BP, (2) chronic stress outside of the office setting related to perceived or actual stigma that could elevate the ambulatory BP, or (3) poor sleep in PWH. Future research should investigate these possibilities.

Third, our study demonstrated that ABPM—and particularly asleep BP measurements—may provide information independent of office BP to explain the relatively higher left ventricular mass in PWH. Large comparative studies have consistently demonstrated that, after adjusting for traditional risk factors including office BP, PWH have higher LVMI than PWoH (19,32,33). However, the underlying cause of the greater left ventricular mass in PWH is not understood. Our data suggest that the relatively higher nocturnal BP observed in PWH could help to explain the previously reported higher left ventricular mass in PWH. In our main analysis, a 10 mmHg higher asleep systolic BP was associated with an ~3.5 g/m^2^ increase in LVMI, even after adjusting for clinic BP and other CVD risk factors. In the first sensitivity analysis, requiring seven asleep BP measurements, a 10 mmHg higher asleep systolic BP was independently associated with an even greater ~5 g/m^2^ increase in LVMI. This difference could be clinically important since studies have shown that each 10 g/m^2^ incremental increase in LVMI is associated with a 33% increased risk of CVD (34). Notably, the fact that we did not observe the same significant association in PWoH may be related to statistical power in this group.

Major strengths of the current study include the large sample size; there were 959 valid ABPM recordings in a large cohort at two time points, enrollment and one month. Unattended, automated office BP was also measured rigorously and consistently at every study visit. Also, multiple sensitivity analyses were performed, demonstrating similar results for more rigorous ABPM quality criteria and for participants with two valid ABPM measurements. Although the population of PWH is similar to other groups of PWH attending clinics in urban and peri-urban sub-Saharan Africa, our results might not be generalizable to other regions, to rural areas, or to adults in high-income countries. Additionally, the lower office BP in relation to ABPM in both groups could have been related to the fact that we used unattended office BP measurement. Further, this is a cross-sectional analysis, limiting our ability to establish temporal, causal relationships between HIV, BP parameters, and left ventricular hypertrophy; future longitudinal results from this cohort are, however, likely to provide additional insight.

## Conclusion

This large comparative cohort study confirms and extends prior cross-sectional studies, demonstrating subtle but potentially clinically important differences in the relationship between asleep BP and awake BP, and office BP in PWH compared to PWoH. These findings suggest that office BP might underestimate the true BP-related CVD risk in PWH. For HIV clinicians, this study suggests that ABPM could be utilized to better guide BP diagnosis and treatment in PWH. Our findings also raise the hypothesis that PWH might benefit from the initiation of BP medications at a lower office BP threshold. As we have recently learned from the REPREIVE trial of early initiation of statins, PWH might benefit from more aggressive diagnostic workup and treatment to prevent CVD (35).

## Data Accessibility Statement

We will make the dataset available to users upon request under a data sharing agreement that includes the following: a commitment to use the data for research purposes and not participant identification, commitment to securing the data, and a commitment to destroying the data after analyses are completed. A data dictionary and analytic code will be provided with any shared data.

## Additional Files

The additional files for this article can be found as follows:

10.5334/gh.1542.s1 Supplementary Material 1. Table S1 and Figure S1.

10.5334/gh.1542.s2 Supplementary Material 2. Sensitivity Analysis 1: Tables S2–S3 and Figures S2–S3.

10.5334/gh.1542.s3 Supplementary Material 3. Sensitivity Analysis 2: Tables S4–S5 and Figure S4.
