# Supplementary material for: Ambulatory Blood Pressure Patterns and Left Ventricular Mass Index in Tanzanian Adults Living with and without HIV
Source: Glob Heart. 2026 Mar 24;21(1):26. doi: 10.5334/gh.1542 (PMC13025247; doi:10.5334/gh.1542)
Supplement: Supplementary Material 1. — Table S1 and Figure S1. [file gh-21-1-1542-s1.pdf]

Ambulatory Blood Pressure Patterns and Left Ventricular Hypertrophy in Tanzanian Adults Living with HIV

SUPPLEMENTARY MATERIAL

Table S1: Dipping categories by HIV and hypertension status

	**Without hypertension**		**Hypertension**	
N (%)	**PWH (N=235)**	**PWoH (N=226)**	**PWH (N=248)**	**PWoH (N=250)**
**Systolic**				
Extreme dipping	1 (0.4)	1 (0.4)	3 (1.2)	3 (1.2)
Dipping	88 (37.4)	93 (41.2)	43 (17.3)	55 (22.0)
Non-dipping	133 (56.6)	129 (57.1)	156 (62.9)	154 (61.6)
Rising	13 (5.5)	3 (1.3)	46 (18.5)	38 (15.1)
**Diastolic**				
Extreme dipping	32 (13.6)	46 (20.4)	19 (7.7)	27 (10.8)
Dipping	136 (57.9)	150 (66.4)	96 (38.7)	119 (47.6
Non-dipping	64 (27.2)	30 (13.3)	114 (46.0)	87 (34.8)
Rising	3 (1.3)	0 (0)	19 (7.7)	17 (6.8)

Figure S1. The relationship between blood pressure parameters and LVMI in PWH (upper panel) vs. PWoH (lower panel)

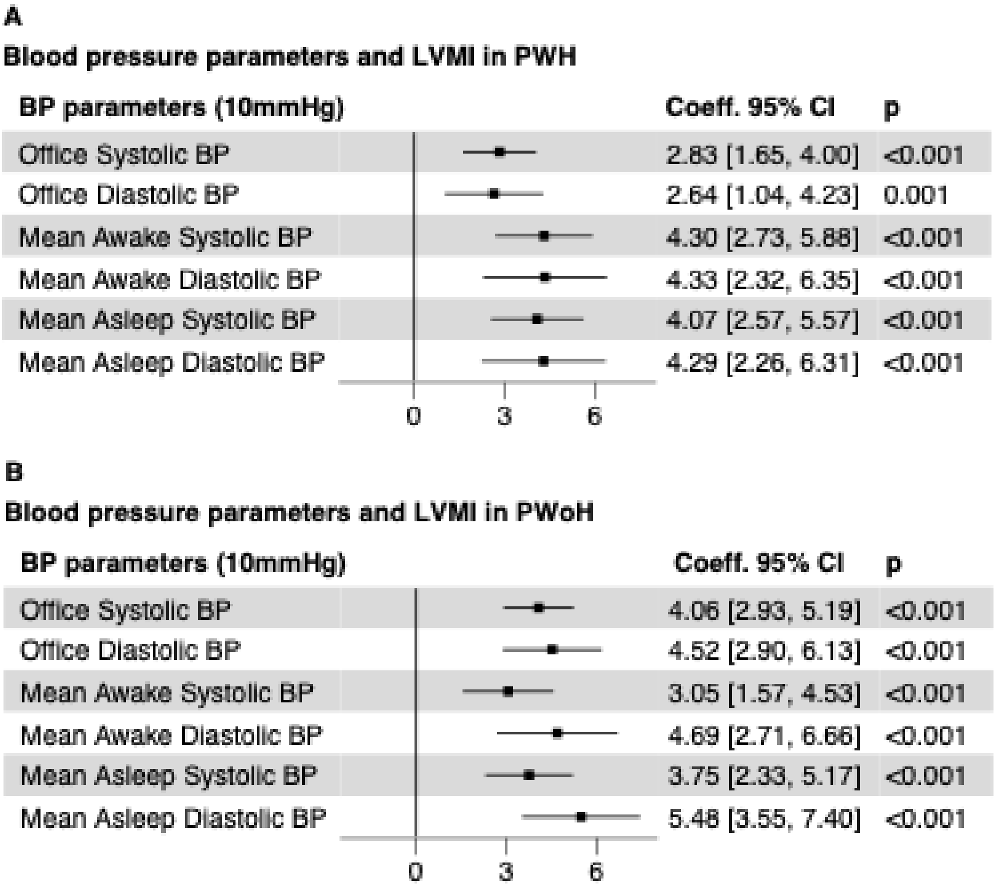

All linear regression models were adjusted for age, sex, tobacco use, taking antihypertensives, and hemoglobin.
