# Supplementary material for: Ambulatory Blood Pressure Patterns and Left Ventricular Mass Index in Tanzanian Adults Living with and without HIV
Source: Glob Heart. 2026 Mar 24;21(1):26. doi: 10.5334/gh.1542 (PMC13025247; doi:10.5334/gh.1542)
Supplement: Supplementary Material 2. — Sensitivity Analysis 1: Tables S2–S3 and Figures S2–S3. [file gh-21-1-1542-s2.pdf]

Sensitivity Analysis 1: 812 participants with 20 valid awake measurements and 7 valid asleep measurements

We conducted a sensitivity analysis according to a stricter definition for ABPM measurement validity (20 valid awake measurements and 7 asleep measurements). Baseline characteristics remained similar for participants included in the sensitivity analysis (**Table S1**). Office BP was higher in PWoH compared to PWH (**Table S2**). Diastolic non-dipping was significantly higher in PWH (IRR 1.52 [1.26-1.83]), but systolic non-dipping was similar by HIV status (**Figure S2**) according to Poisson regression. Mean asleep BP was strongly associated with LVMI among PWH with hypertension, but the strength of association between ABPM parameters and LVMI weaker among PWH who did not have hypertension (**Figure S3**).

Table S2. Baseline characteristics of 812 participants included in Sensitivity Analysis 1

	**PWH (N=404)** Median [25-75 percentiles] / N (%)	**PWoH (N=408)** Median [25-75 percentiles] / N (%)
**Age**	43 [39-50]	43 [36-50]
**Female sex**	278 (68.8)	275 (67.4)
**Education level**		
Primary school or less	342 (84.7)	310 (76.0)
Complete secondary school	49 (12.1)	77 (18.9)
University/college	13 (3.2)	21 (5.1)
**Low income** (<$1.90 USD/day)	232 (57.4%)	240 (58.8%)
**Mode of transport**		
Private vehicle	49 (12.1)	52 (12.7)
Public transport	216 (53.5)	209 (51.2)
Walking/cycling	139 (34.4)	147 (36.0)
**Manual labor**	131 (32.4)	141 (34.6)
**Current tobacco use**	16 (4.0)	31 (7.6)
**Current alcohol use**	122 (30.2)	121 (29.7)
**Taken antihypertensive medication in the past week**	6 (1.5)	11 (2.7)
**BMI**		
Underweight (<18.5 kg/m^2)	45 (11.1)	50 (12.3)
Normal (18.5-24.9 kg/m^2)	229 (56.7)	217 (53.2)
Overweight/Obese (≥25 kg/m^2)	130 (32.2)	141 (34.6)
**Diabetes**	6 (1.5)	4 (1.0)
**Waist circumference** (cm)	82.9 [75.3-93.0]	83.2 [76.7-93.6]
**Hemoglobin** (g/dl)	13.3 [11.4-14.8]	13.7 [12.5-15.0]
**CD4+ T-cell count** (cells/m^3)	717 [539-953.5]	N/A

Table S3: Blood pressure parameters for 812 participants included in Sensitivity Analysis 1

	**PWH (N=404)** Mean (SD)	**PWoH (N=408)** Mean (SD)	**Adjusted Difference**	**p-value**
BP parameters from office and ambulatory BP measurements				
**Office systolic BP** (mmHg)	113 (16.3)	116 (16.2)	-2.9 [-5.0, -0.7]	0.012
**Office diastolic BP** (mmHg)	68.9 (12.2)	71.3 (11.1)	-1.6 [-3.2, -0.1]	0.004
**Awake mean systolic BP** (mmHg)	122 (12.4)	123 (12.3)	-1.4 [-3.0, 0.3]	0.072
**Awake mean diastolic BP** (mmHg)	80.3 (10.2)	81.7 (9.4)	-0.9 [-2.1, 0.4]	0.045
**Asleep mean systolic BP** (mmHg)	114 (12.7)	114 (12.4)	0.4 [-1.3, 2.1]	0.666
**Asleep mean diastolic BP** (mmHg)	70.9 (9.7)	70.4 (9.4)	0.6 [-0.7, 1.9]	0.422
**Systolic nocturnal dipping %**	-6.3 (5.5)	-7.7 (5.7)	1.4 [0.6, 2.2]	<0.001
**Diastolic nocturnal dipping %**	-11.5 (6.8)	-13.8 (6.9)	1.7 [0.7, 2.6]	<0.001
	**PWH (N=404)** Mean (SD)	**PWoH (N=408)** Mean (SD)	**Unadjusted Difference**	**p-value**
Within-participant differences between ambulatory and office BP measurements				
**Awake mean systolic BP vs. office BP difference** (mmHg)	8.5 (9.7)	7.2 (10.1)	1.3 [-0.04, 2.7]	0.058
**Awake mean diastolic BP vs. office BP difference** (mmHg)	11.4 (7.5)	10.4 (6.5)	1.0 [0.04, 2.0]	0.041
**Asleep mean systolic BP vs. office BP difference** (mmHg)	0.8 (11.4)	-2.4 (11.7)	3.3 [1.7, 4.8]	<0.001
**Asleep mean systolic BP vs. office BP difference** (mmHg)	2.1 (8.4)	-0.9 (7.7)	2.9 [1.8, 4.0]	<0.001

*This table displays the relationship between BP parameters and HIV status both with raw averages and adjusted differences for BP parameters and unadjusted differences for within-participant differences.

Figure S2. Systolic and diastolic non-dipping by HIV and hypertension status according to Sensitivity Analysis 1

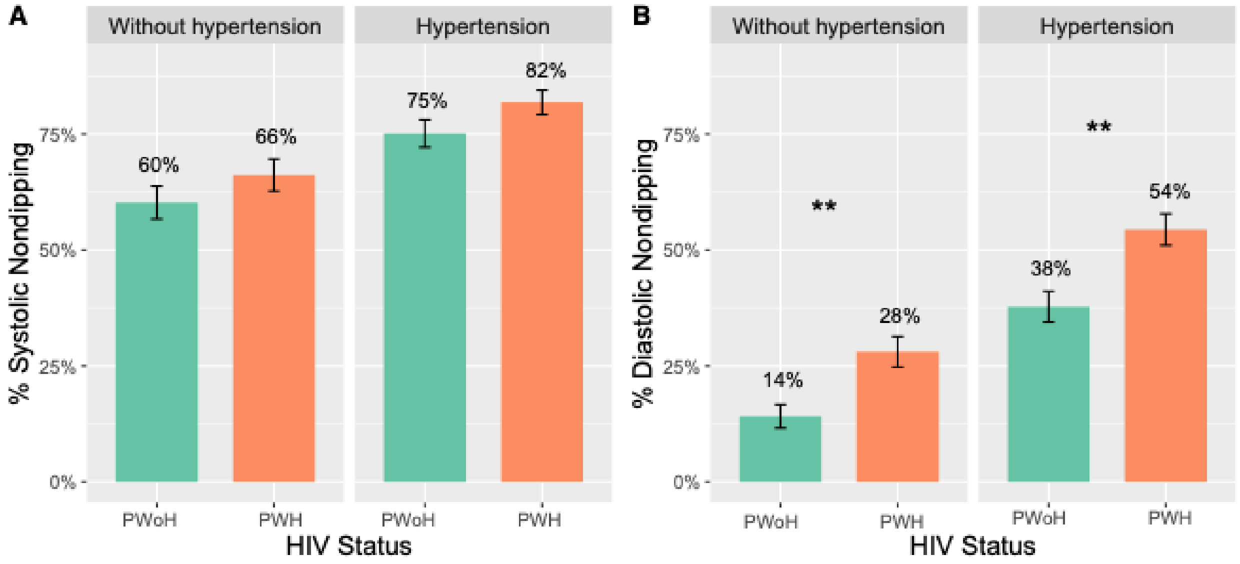

** indicates p-values less than 0.01.

Figure S3. The relationship between ABPM parameters and LVMI by HIV and hypertension status according to Sensivity Analysis 1

BP parameters (10mmHg)	Subset	Adj. Coeff (95% CI)	p
Mean Asleep Systolic BP	HIV, HTN	4.81 [2.00, 7.62]	<0.001
Mean Asleep Systolic BP	HIV, no HTN	3.64 [−0.80, 8.09]	0.108
Mean Asleep Systolic BP	no HIV, HTN	1.81 [−1.20, 4.82]	0.237
Mean Asleep Systolic BP	no HIV, no HTN	−1.00 [−5.04, 3.03]	0.624
Mean Awake Systolic BP	HIV, HTN	3.41 [0.01, 6.81]	0.050
Mean Awake Systolic BP	HIV, no HTN	1.95 [−2.61, 6.52]	0.400
Mean Awake Systolic BP	no HIV, HTN	−0.96 [−4.28, 2.36]	0.568
Mean Awake Systolic BP	no HIV, no HTN	−0.77 [−4.77, 3.23]	0.705

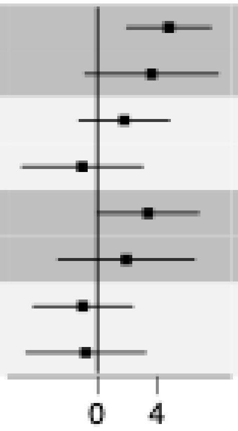

Adjusted coefficients (“Adj Coeff”) were calculated using as the regression coefficient for each ABPM parameter in each stratum in models adjusted for office systolic BP and traditional CVD risk factors including age, sex, BMI, education, tobacco use, alcohol use, and antihypertensive medication use. In the first row, for example, among people with HIV and hypertension, a 10 mmHg increase in mean asleep systolic BP was associated with a 4.8 g/m^2 increase in LVMI.
