# Supplementary material for: Ambulatory Blood Pressure Patterns and Left Ventricular Mass Index in Tanzanian Adults Living with and without HIV
Source: Glob Heart. 2026 Mar 24;21(1):26. doi: 10.5334/gh.1542 (PMC13025247; doi:10.5334/gh.1542)
Supplement: Supplementary Material 3. — Sensitivity Analysis 2: Tables S4–S5 and Figure S4. [file gh-21-1-1542-s3.pdf]

Sensitivity Analysis 2: 936 participants who were not taking antihypertensives (≥70% successful recordings with ≥3 at night)

We conducted a second sensitivity analysis exclusive of participants taking antihypertensive medication (≥70% successful records with ≥3 at night). Baseline characteristics remained similar for participants included in this sensitivity analysis (**Table S3**). Office BP was higher in PWoH compared to PWH (**Table S4**). Mean asleep BP was strongly associated with LVMI among PWH with hypertension and without hypertension (**Figure S5**).

Table S4. Baseline characteristics of 936 participants included in Sensitivity Analysis 2

	**PWH (N=474)** Median [25-75 percentiles] / N (%)	**PWoH (N=462)** Median [25-75 percentiles] / N (%)
**Age** (median [IQR])	46 [39-50]	42 [36-49]
**Female sex** (%)	333 (70.3)	318 (68.8)
**Education level** (%)		
Primary school or less	404 (85.2)	351 (76.0)
Complete secondary school	57 (12.0)	88 (19.0)
University/college	13 (2.7)	23 (5.0)
**Low income** (<$1.90 USD/day) (%)	273 (57.6)	260 (56.3)
**Mode of transport** (%)		
Private vehicle	68 (14.3)	55 (11.9)
Public transport	249 (52.5)	233 (50.4)
Walking/cycling	157 (33.1)	174 (37.7)
**Manual labor** (%)	155 (32.7)	151 (32.7)
**Current tobacco use** (%)	20 (4.2)	35 (7.6)
**Current alcohol use** (%)	148 (31.2)	136 (29.4)
**Taken antihypertensive medication in the past week** (%)	0 (0.0)	0 (0.0)
**BMI** (%)		
Underweight (<18.5 kg/m^2)	45 (9.5)	37 (8.0)
Normal (18.5-24.9 kg/m^2)	270 (57.0)	243 (52.6)
Overweight/Obese (≥25 kg/m^2)	159 (33.5)	182 (39.4)
**Diabetes** (%)	9 (1.9)	4 (0.9)
**Waist circumference** (cm)	83.1 [75.5-93.3]	83.2 [76.4-93.4]
**Hemoglobin** (g/dl)	13.3 [11.6-14.8]	13.7 [12.5-15.0]
**CD4+ T-cell count** (cells/m^3)	716 [537-944]	N/A

Table S5: Blood pressure parameters for 936 participants included in Sensitivity Analysis 2

	**PWH (N=474)** Mean (SD)	**PWoH (N=462)** Mean (SD)	**Adjusted Difference**	**p-value**
BP parameters from office and ambulatory BP measurements				
**Office systolic BP** (mmHg)	113 (16.0)	116 (16.5)	-2.8 [-4.8, -0.9]	0.004
**Office diastolic BP** (mmHg)	68.8 (12.0)	71.0 (10.8)	-1.7 [-3.1, -0.3]	0.017
**Awake mean systolic BP** (mmHg)	122 (12.1)	123 (11.9)	-1.5 [-3.0, 0.0]	0.054
**Awake mean diastolic BP** (mmHg)	80.1 (10.0)	81.3 (9.1)	-0.9 [-2.1, 0.3]	0.131
**Asleep mean systolic BP** (mmHg)	114 (12.6)	114 (11.0)	-0.3 [-1.8, 1.3]	0.748
**Asleep mean diastolic BP** (mmHg)	70.5 (9.6)	70.3 (9.3)	0.1 [-1.1, 1.3]	0.830
**Systolic nocturnal dipping %**	-6.5 (5.6)	-7.5 (5.6)	0.9 [0.2, 1.6]	0.015
**Diastolic nocturnal dipping %**	-11.7 (6.9)	-13.5 (6.9)	1.2 [0.3, 2.1]	0.007
	**PWH (N=474)** Mean (SD)	**PWoH (N=462)** Mean (SD)	**Unadjusted Difference**	**p-value**
Within-participant differences between ambulatory and office BP measurements				
**Awake mean systolic BP vs. office BP difference** (mmHg)	8.5 (9.4)	7.5 (10.0)	1.0 [-0.3, 2.2]	0.127
**Awake mean diastolic BP vs. office BP difference** (mmHg)	11.3 (6.6)	10.3 (6.6)	1.0 [0.1, 1.9]	0.030
**Asleep mean systolic BP vs. office BP difference** (mmHg)	0.5 (11.1)	-1.8 (11.5)	2.3 [0.9, 3.8]	0.002
**Asleep mean systolic BP vs. office BP difference** (mmHg)	1.8 (8.3)	-0.7 (7.7)	2.4 [1.5, 3.5]	<0.001

*This table displays the relationship between BP parameters and HIV status both with raw averages and adjusted differences for BP parameters and unadjusted differences for within-participant differences.

Figure S4. Systolic and diastolic non-dipping by HIV and hypertension status according to Sensitivity Analysis 2

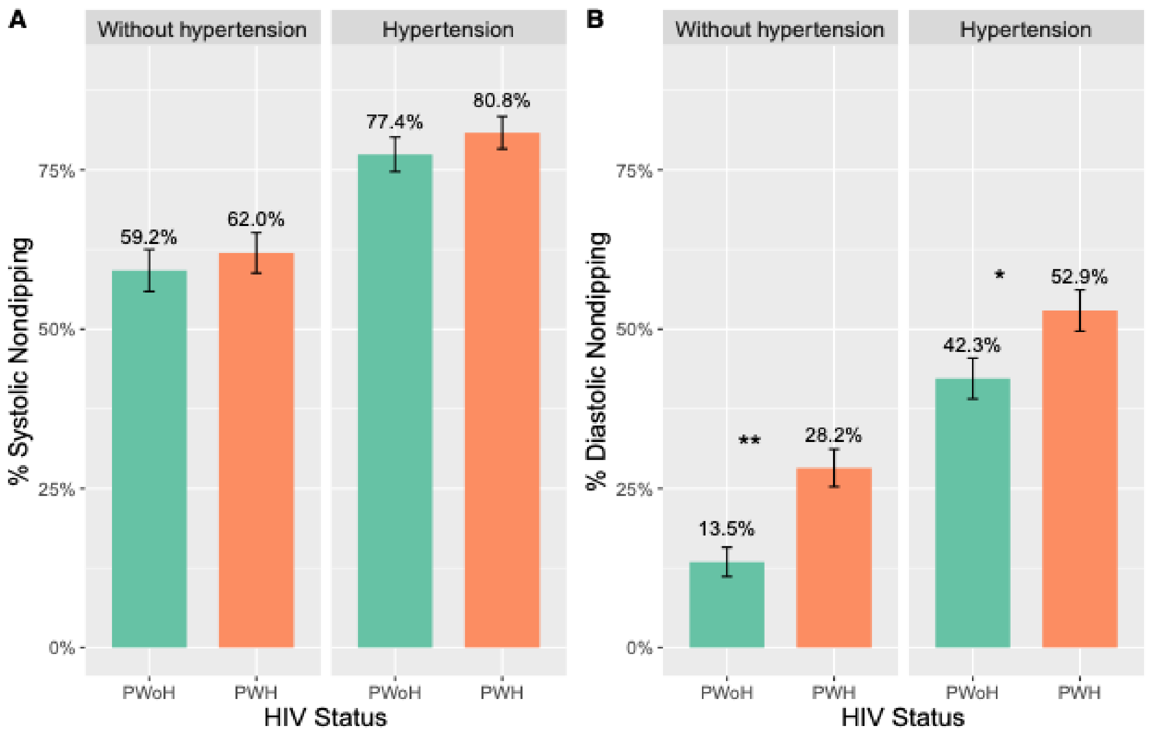

** indicates p-values less than 0.01; * indicates p-value less than 0.05.

Figure S5. The relationship between ABPM parameters and LVMI by HIV and hypertension status according to Sensivity Analysis 2

BP parameters (10mmHg)	Subset	Adj. Coeff [95% CI]	p
Mean Asleep Systolic BP	HIV, HTN	3.40 [0.53, 6.27]	0.021
Mean Asleep Systolic BP	HIV, no HTN	3.90 [0.08, 7.72]	0.045
Mean Asleep Systolic BP	no HIV, HTN	0.81 [−2.15, 3.76]	0.591
Mean Asleep Systolic BP	no HIV, no HTN	0.44 [−3.06, 3.93]	0.805
Mean Awake Systolic BP	HIV, HTN	2.72 [−0.63, 6.07]	0.111
Mean Awake Systolic BP	HIV, no HTN	3.10 [−1.08, 7.28]	0.145
Mean Awake Systolic BP	no HIV, HTN	−2.04 [−5.15, 1.08]	0.199
Mean Awake Systolic BP	no HIV, no HTN	−0.39 [−3.81, 3.03]	0.822

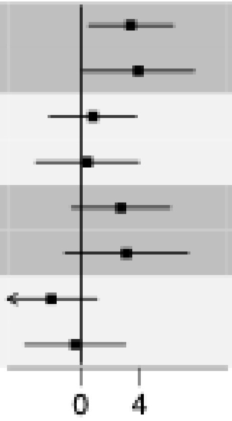

Adjusted coefficients ("Adj Coeff") were calculated using as the regression coefficient for each ABPM parameter in each stratum in models adjusted for office systolic BP and traditional CVD risk factors including age, sex, BMI, education, tobacco use, alcohol use, and antihypertensive

medication use. In the first row, for example, among people with HIV and hypertension, a 10 mmHg increase in mean asleep systolic BP was associated with a 3.4 g/m^2 increase in LVMI.
